# Leader-Inspired Nutrition: Promoting Safe Dietary Supplement Use for Service Members

**DOI:** 10.3390/nu17223592

**Published:** 2025-11-17

**Authors:** Andrea T. Lindsey, Cindy Crawford, Tanisha L. Currie, Mary McCarthy, Patricia A. Deuster

**Affiliations:** 1 Consortium for Health and Military Performance, Department of Military and Emergency Medicine, F. Edward Hébert School of Medicine, Uniformed Services University, Bethesda, MD 20814, USA; cindy.crawford.ctr@usuhs.edu (C.C.); pdeuster@me.com (P.A.D.); 2 Henry M. Jackson Foundation for the Advancement of Military Medicine, Inc., Bethesda, MD 20817, USA; 3 Center for Enabling Capabilities, Walter Reed Army Institute of Research, Fort Glen Annex, Silver Spring, MD 20910, USA; tanisha.l.currie.mil@health.mil; 4 Madigan Army Medical Center, Joint Base Lewis, Tacoma, WA 98431, USA; mary.s.mccarthy1.civ@health.mil

**Keywords:** decision making, dietary supplements, energy drinks, leader-inspired nutrition, leadership, military personnel, nutrition, performance products, safety

## Abstract

Military readiness and performance are critical to national defense. Service Members must maintain their health and fitness to remain deployable. Dietary supplement use in the military is prevalent and remains a significant concern due to rampant misinformation, product adulteration, and documented adverse events. In March 2022, the Department of Defense issued Instruction (DoDI) 6130.06: *Use of Dietary Supplements in the DoD*, formally establishing Operation Supplement Safety (OPSS) [1] as the program of record for everything related to dietary supplements. Leaders, as role models within their organizations, can serve as facilitators in promoting the safe and informed use of dietary supplements in the military through an innovative framework called Leader-Inspired Nutrition (LIN). LIN is a leadership-driven strategy aimed at enhancing Service Members’ health, encompassing seven pillars focused on nutritional fitness, including the informed and safe use of dietary supplements. This paper details how leaders can engage with Service Members and advocate for the safe use of dietary supplements by utilizing five strategic imperatives, educational initiatives, and resources provided by OPSS.

## 1. Introduction

Dietary supplement use is prevalent in the military, with approximately 74% of U.S. Service Members regularly consuming them for various reasons, including performance enhancement, weight loss, muscle gain, and overall health [2]. The market is saturated with thousands of products, some of which lack scientific evidence to support their purported safety or efficacy [3]. Therefore, when supplementation is necessary or desired, Service Members must have easy access to reliable products. Unfortunately, the use of certain dietary supplements may lead to adverse events that compromise readiness, performance, and health, rather than enhance them. Well-documented reports detail Service Members experiencing adverse events, some mild and some serious, including death, following dietary supplement use [4,5,6,7,8]. Beyond health and performance impacts, some dietary supplements may contain prohibited substances, either listed on the label or undisclosed, which can negatively affect a Service Member’s career or retention status [9,10,11,12,13,14,15,16,17,18]. Furthermore, the ongoing financial commitment of consistently purchasing these supplements may strain Service Members’ personal budgets, given the unverified or inconsistent assurances of efficacy or safety [19].

In 2012, the Assistant Secretary for Health Affairs requested a Department of Defense (DoD)-wide educational campaign on the safe use of dietary supplements following numerous adverse events and several deaths associated with dietary supplements containing 1,3-dimethylamylamine (DMAA), a stimulant similar to amphetamine [20]. In response to this request, the DoD Dietary Supplement and Other Self-Care Products Subcommittee, the U.S. Army Public Health Command, and the Consortium for Health and Military Performance at the Uniformed Services University launched Operation Supplement Safety (OPSS), a DoD-wide educational initiative aimed at increasing awareness across the DoD community about potential health risks and how to choose safe dietary supplements [21].

In March 2022, DoD issued DoD Instruction (DoDI) 6130.06: *Use of Dietary Supplements in the DoD*, which formalized OPSS as the mandated program of record for everything related to dietary supplements [1]. The DoDI states that OPSS will maintain the official *DoD Prohibited Dietary Supplement Ingredients List* and provide evidence-based education on dietary supplements. The policy applies to “OSD *[Office of the Secretary of Defense]*, the Military Departments, the Office of the Chairman of the Joint Chiefs of Staff and the Joint Staff, the Combatant Commands, the Office of the Inspector General of the Department of Defense, the Defense Agencies, the DoD Field Activities, and all other organizational entities within the DoD.” (1, page 3) The DoDI mandates education and training for all Service Members and those who provide health-related services to military members. This requirement includes healthcare personnel, health promotion specialists, fitness leaders, athletic trainers, health promotion personnel, and strength and conditioning specialists. The mission of OPSS is to provide the most reliable evidence-based information on dietary supplements, including energy drinks and other health, wellness, and performance products. When reliable scientific evidence on dietary supplement ingredients (such as emerging research on creatine) becomes available, that information must be accurately reflected across the entire digital platform, including the public-facing website. OPSS educates Service Members, retirees, their families, leaders, healthcare providers, and DoD civilians to support informed decision making regarding these products [21].

The dietary supplement market continues to experience significant growth, accompanied by marketing practices that often target Service Members. These practices may encompass exaggerated claims regarding rapid weight loss, muscle gain, and enhanced energy and performance, as well as offering military discounts as a tactic to entice Service Members [22]. Service Members may choose to use dietary supplements to address their health and performance goals. It is important that they have access to safe products and know how to mitigate risks. The landscape of dietary supplements has undergone significant changes since the passage of the Dietary Supplement Health and Education Act (DSHEA) in 1994 [23]. Under DSHEA, dietary supplements are regulated as a subcategory of food, rather than as drugs, and are defined as products intended to supplement the diet. Manufacturers are responsible for ensuring the safety and accurate labeling of their products. The Food and Drug Administration (FDA) can only take regulatory action after a product is on the market and shown to be unsafe, misbranded, or adulterated. Although DSHEA permits general structure/function claims, such as “supports a healthy immune function,” some performance-enhancing dietary supplements may violate these regulations by making misleading or illegal drug-like claims. Examples of potentially misleading claims on dietary supplement labels include the following:

- *“Natural anabolic agent for explosive strength and power”*
- *“Clinically proven fat burner for healthy and safe weight loss”*
- *“Uniquely effective at relieving pain & enhancing mood”*

Beyond potentially misleading claims, dietary supplements are sometimes mislabeled, contaminated, or adulterated, with some products containing substances that are not legally permitted in dietary supplements [9,24,25]. For example, certain performance-enhancing dietary supplements, particularly those promising dramatic results such as rapid weight loss or extreme muscle gain, have been found to contain undeclared substances, including anabolic steroids, unapproved stimulants, or prescription-only drugs [15,25]. In fact, the FDA has warned consumers that many products marketed for bodybuilding, weight loss, sexual enhancement, arthritis and joint pain, diabetes, and sleep aids may be contaminated with dangerous hidden ingredients that “pose a serious health risk and are not guaranteed to work” [17,25]. More recently, a new category of novel or experimental substances has emerged in the marketplace. These unapproved drugs and research chemicals are often disguised as dietary supplements, but labeled “for research use only” or “not for human consumption,” despite being accessible and marketed to consumers [26]. These substances pose serious risks to Service Members and represent a Total Force readiness issue [15,26]. A positive drug test, performance decrement, or adverse event can ruin a Service Member’s career. Despite these health and performance risks, performance enhancement supplements remain widely used within the military [6,27].

## 2. Leader-Inspired Nutrition for Promoting Safe Supplement Use

Leaders are crucial as both role models and advocates for promoting the safe use of dietary supplements in the military, and their leadership is essential in mitigating these risks. Empowering leaders to engage in productive discussions about nutritional fitness, including the safe use of dietary supplements, and to model healthy behaviors, can facilitate optimal performance and readiness across the Force.

Leader-Inspired Nutrition (LIN) is a leadership-driven strategy that promotes the conscious advancement of Service Members’ health and well-being. The LIN framework emphasizes the leader’s role in cultivating a culture of health by encouraging healthy behaviors and sound nutritional practices within the organization [28]. LIN was initially developed by a core group of scientists with a vested interest in improving the military nutrition environment. The framework comprises seven strategic pillars, each designed to address nutrition-related challenges that affect recruitment, retention, and overall readiness. These pillars include integrating Nutrition 101, integrating Dietary Supplements 101, promoting a performance-focused food environment, modeling top–down nutrition behaviors, considering economic factors that influence nutrition choices, promoting the utilization of DoD and partners’ wellness resources, and evaluating the impact of Total Force Fitness.

This article expands on the conceptual framework of LIN [28,29], focusing specifically on the integration of dietary supplement education, referred to as “Dietary Supplements 101,” and the important role of leadership in promoting informed and safe dietary supplement use. Leadership support is essential for promoting safe supplement use, protecting operational readiness, and preventing adverse outcomes. Leaders can inspire and support Service Members through implementing five key strategic imperatives: (I) Provide Education and Training Opportunities, (II) Disseminate Key Messages on Dietary Supplement Safety, (III) Model and Reinfoce Responsible Dietary Supplement Use, (IV) Assess the Dietary Supplement Environment on Base, and (V) Encourage All Personnel to Use the OPSS Website at OPSS.org (https://www.opss.org/ (accessed on 21 October 2025)). Given the prevalence of dietary supplement use in the military, along with potential for contaminated and/or adulterated products, the implementation of LIN in the context of dietary supplements is both necessary and timely.

## 3. Key Strategic Imperatives

### 3.1. Strategic Imperative I: Provide Education and Training Opportunities as Required by DoDI 6130.06

To effectively advocate for and promote the informed use of dietary supplements, including energy drinks and other health, wellness, and performance products, leaders must first be educated and well-informed themselves. Leaders can participate in education and training events delivered by OPSS and gain the necessary knowledge to help guide Service Members. Leaders can encourage all subordinates to attend educational events and provide the time and space needed for all Service Members to learn about the safe use of dietary supplements [21]. In line with LIN principles, leaders should emphasize the importance and purpose of nutrition and dietary supplement education.

### 3.2. Strategic Imperative II: Disseminate Key Messages on Dietary Supplement Safety

Leaders can effectively disseminate crucial dietary supplement information [30]. Some key messages of dietary supplement, health, wellness, and performance product safety for leaders to know and disseminate are:

- FDA does not evaluate dietary supplement products for safety, quality, or effectiveness before they enter the market. Dietary supplements are intended to supplement the diet. They are not substitutes for a healthy, balanced eating plan.
- According to DoDI 6130.06, Service Members are not allowed to use dietary supplements with prohibited ingredients.
- Dietary supplements may contain ingredients not listed on the Supplement Facts label.
- If Service Members choose to use a dietary supplement, they should look for products that carry a reputable third-party certification seal, such as Banned Substances Control Group (BSCG) Certified Drug Free, LGC’s Informed Sport, National Sanitation Foundation International (NSF) Certified Sport, and United States Pharmacopeia (USP) verified.
- Third-party certification does not guarantee a dietary supplement is safe or effective, but will ensure the product contains what is listed on the label, thereby reducing the risk of consuming potentially unsafe or prohibited ingredients and reducing the chance of a positive DoD drug test.

### 3.3. Strategic Imperative III: Model and Reinforce Responsible Dietary Supplement Use

Leaders can encourage responsible dietary supplement use by modeling and promoting the responsible use of dietary supplements and other health, wellness, and performance products. The leader can be attentive and incorporate “leadership walk-rounds” into day-to-day activities.

- Do performance dietitians or other health professionals have concerns that can be addressed during these rounds?
- What do Service Members need to achieve their personal health goals and maintain overall well-being?
- How do they assist Service Members with questions about dietary supplements and performance products?
- Do they know where to go for credible, evidence-based information?

Leaders should be aware of sources of misinformation, such as unethical marketing practices or unvalidated peer advice. The leader can emphasize during training that only science-based recommendations from experts are reliable. Leaders should encourage Service Members to seek guidance from a healthcare professional if they want to consider a dietary supplement, and utilize resources on OPSS.org.

### 3.4. Strategic Imperative IV: Assess the Dietary Supplement Environment on Base

In line with modeling and emphasizing responsible use, leaders can be aware of the dietary supplement environment on base.

- Are there posters and advertisements that are misleading?
- Is there an opportunity for OPSS educational materials to be displayed to help Service Members choose safe supplements? Is there an opportunity to engage with the on-base retail establishments to have them display OPSS resources?
- Is there an opportunity to engage OPSS with leaders on base to ensure resources are distributed?
- Leaders can work with OPSS, other leaders, and on-base retail establishments to assess whether a sufficient number of dietary supplement products available on store shelves have been third-party certified by organizations such as BSCG Certified Drug Free, LGC’s Informed Sport, or NSF Certified Sport.

Currently, no regulatory authority mandates or conducts quality reviews of dietary supplements before they are sold. Therefore, the only way to verify a product’s actual ingredients is through testing by a reputable, independent, third-party organization [31,32]. Third-party certification by organizations as listed above also tests for contaminants such as drugs and other substances not permitted in dietary supplements. Leaders can help promote the availability of third-party certified products on base. Without this, leaders cannot guarantee Service Members have access to safe dietary supplements on base. OPSS continues to emphasize the importance of third-party certification to minimize risks to Service Members and the Force.

Furthermore, access to an environment with potentially unsafe dietary supplements or those with questionable claims may negatively impact Service Members’ financial fitness. They might spend large sums of money on dietary supplements with a questionable return on investment, rather than on nutritious foods to fuel their performance. A leader should, therefore, consider the economic implications of dietary supplement use that could lead to financial strain or unnecessary hardship for Service Members [29,33].

### 3.5. Strategic Imperative V: Encourage All Personnel to Use the OPSS Website at OPSS.org

Leaders should encourage all personnel to use the OPSS.org website for evidence-based information about dietary supplements, health, wellness, and performance products. Posters and signage about resources and tools available at OPSS.org are accessible and provide appealing infographics for team members to display. These should be visible near or in the shops on base, at fitness centers on base, and other establishments selling dietary supplements, so Service Members can be aware of the resources available for making informed use decisions.

#### 3.5.1. What Resources and Tools Can Leaders Leverage?

- *Evidence-based content:* OPSS produces a variety of evidence-based content, including articles, videos, handouts, and infographics, all publicly available on OPSS.org. Content development priorities are guided by input from the Services, inquiries from Service Members via the Ask the Expert portal, working partnerships, and market analyses of trending dietary supplement ingredients. The OPSS Team monitors and investigates potentially dangerous products and ingredients marketed as dietary supplements. Additionally, OPSS evaluates the efficacy and safety of dietary supplement ingredients to optimize performance and promote overall health. The resulting information is used to develop evidence-based educational materials and services, empowering Service Members to make informed and safe dietary supplement decisions.
- *DoD Prohibited Dietary Supplement Ingredients List:* In accordance with DoDI 6130.06, the OPSS website provides a database of dietary supplement ingredients prohibited by the DoD to help Service Members know what to avoid when considering dietary supplement products [16]. The database is updated quarterly, or when an FDA action occurs or new scientific information becomes available regarding dietary supplement ingredients. The OPSS Team creates content and resources to raise awareness of prohibited ingredients and continually strives to improve search functionality for all prohibited ingredients and their “other names” that may appear on product Supplement Facts labels.
- *The OPSS Scorecard:* The Scorecard, or “algorithm,” was created to assist all consumers in making an informed decision about dietary supplement products [34]. It guides users through a rapid screening process, promoting a thorough review of product labels before making a purchase. By answering seven “yes” or “no” questions on topics related to third-party certification, multiple ingredient combinations, proprietary blends, and questionable claims, Service Members can determine whether a product might be risky. However, the initial step should always involve checking the *DoD Prohibited Dietary Supplement Ingredients List* [16]. If a product label lists a prohibited ingredient, there is no reason to score the product, as Service Members should not purchase such products.
- *Ask the Expert:* Service Members, family members, leaders, providers, Veterans, allied health professionals, and all consumers can visit the Ask the Expert portal on OPSS.org to submit questions about anything related to dietary supplements and performance products and receive evidence-based responses from the OPSS Team within two weeks. All information is treated as confidential [35].

#### 3.5.2. Presentations and Trainings

Dietary supplement education and training are critical to sustaining a healthy, informed, and prepared military workforce. According to DoDI 6130.06, dietary supplement education is required for all Service Members and those who provide health-related services, as well as healthcare professionals, including DoD military, civilian, and contract providers. The OPSS Team coordinates dietary supplement education efforts across the DoD community for providers, all allied health professionals, and Service Members. The Team delivers both in-person and virtual presentations and trainings, ensures staff representation at health fairs and on military installations, and offers a “Train the Trainer” program for health professionals to extend the reach and impact of education efforts.

#### 3.5.3. Outreach

Spreading the word on evidence-based dietary supplement use is imperative toward ensuring consistent messaging. The OPSS team leverages various social media platforms, including Facebook, Instagram, X/Twitter, and YouTube, to expand efforts to reach Service Members and the DoD community more effectively. OPSS runs a social media campaign biannually, which focuses on increasing awareness about dietary supplements, performance products, and energy drinks. Leadership engagement is critical for the greatest impact.

#### 3.5.4. Partnerships

OPSS partners with other DoD organizations, federal agencies, and professional associations to provide the DoD community with the best information and resources about safe dietary supplement use. Select partners include the Office of the Under Secretary for Personnel and Readiness, Drug Demand Reduction Program, Food and Drug Administration, Federal Trade Commission, National Institutes of Health, Office of Dietary Supplements, National Center for Complementary and Integrative Health, U.S. Special Operations Command, U.S. Department of Agriculture, Department of Justice, and Drug Enforcement Administration. Importantly, OPSS also works closely with local dietitians and health promotion and wellness program staff located on bases, who can help leaders promote LIN and safe supplement use. Most recently, and due to the expanding market of dietary supplements and other dangerous products available to consumers, OPSS, in collaboration with Major League Baseball and U.S. Anti-Doping Agency, has established the Performance Enhancing Substances Consortium to help protect Service Members, athletes, and all consumers from harmful substances in products that could threaten health and careers [26]. Individuals of all ages are seeking performance-enhancing substances at an increasing rate, motivated by a desire to improve both performance and appearance. Many of these individuals may not seek guidance from a healthcare professional or support when experiencing an adverse event from use [36,37,38,39]. Consequently, the framework and approaches for LIN in promoting safe supplement use can be applied not only in the military, but also in other organizational settings where leaders, mentors, and educators can significantly influence the views and behaviors of their employees, athletes, and students.

## 4. Discussion

**Figure 1:**
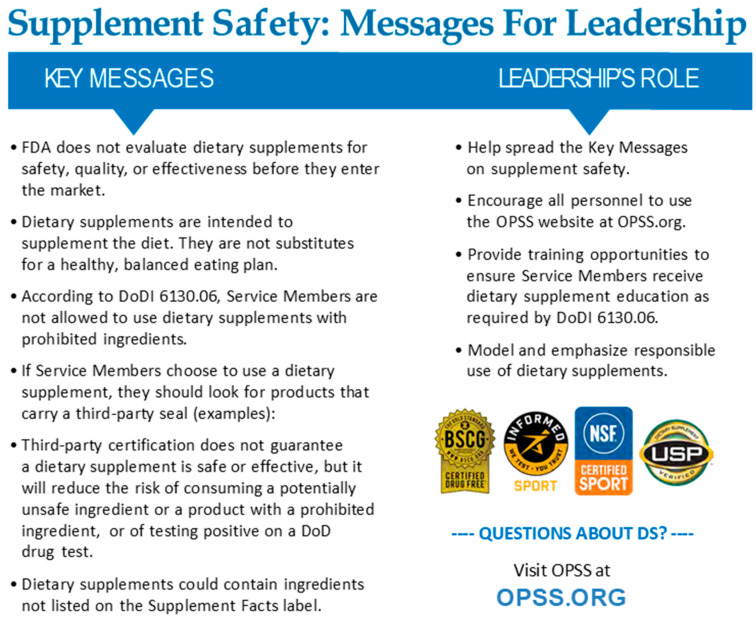
Key Messages for Leadership.

The role of leaders is critical in the space of dietary supplements, health, wellness, and performance products. By implementing key strategic imperatives, leaders can help to empower Service Members to make informed decisions about these products. Certain dietary supplements may be beneficial for optimizing health, well-being, and performance. However, given the current landscape and deceptive marketing of dangerous products, it is imperative to equip Service Members with evidence-based, accurate, and timely information. This directly impacts readiness, retention, and overall health and wellness. The adoption and implementation of the LIN concept could be foundational to health promotion activities across all military environments, ultimately leading to optimized performance for the total Force (Figure 1).

The DoDI 6130.06, *Use of Dietary Supplements in the DoD*, assigns responsibilities and provides procedures for dietary supplement education and use throughout the DoD, at various levels of leadership. It also states, *“The Combatant Commanders with geographic areas of responsibility monitor compliance with the execution of this issuance [for the DoDI] and have overall responsibility for safe use of dietary supplements for all forces assigned or attached to their respective commands, including during military operations.”* OPSS is defined in the DoDI as “*The DoD program dedicated to educating Service members and their families on all aspects of dietary supplements. It includes an educational interactive website supported by Service member education, consultations, and training on dietary supplements, and a research agenda”* [1]. The resources and tools are available and accessible. Leadership support is needed to raise awareness and help spread the messaging through providing training opportunities, modeling and emphasizing responsible use of dietary supplements, assessing the military environment, and encouraging all personnel to use OPSS evidence-based resources.

Service Members use dietary supplements more frequently than the average consumer. Whether choosing whole foods or dietary supplements, Service Members are making important decisions that directly affect their health and performance. Therefore, they must understand how to optimally fuel their bodies and when it may be appropriate to consider a safe, evidence-based, and third-party tested dietary supplement to supplement their diet and support mission readiness. The foundation of informed choices lies in education and consultation with qualified experts, such as Registered Dietitians or healthcare providers, who can address health and performance questions. As a general principle, whole food nutrition should be prioritized to meet individual requirements for health, performance, and overall well-being, with dietary supplements considered when overall nutrition cannot be met through food alone.

## 5. Conclusions

Leaders are at the forefront of mission readiness, which begins with taking care of their people so that the team is fully prepared to execute the mission. As role models, leaders are uniquely positioned to influence Service Members’ health-related choices and drive positive change across the force. Dietary supplement use is widespread in the military, and while some products may support health and performance, others pose potential risks to Service Members’ well-being and careers. To mitigate these risks, it is essential to continuously provide Service Members with the education, tools, and resources needed to make informed decisions about dietary supplement use. By adopting and implementing one or more strategic imperatives focused on informed and safe use of dietary supplements, leaders can strengthen the operational environment and foster a culture of health, resilience, and readiness.

## Data Availability

No new data were created or analyzed in this study. Data Sharing is not applicable to this article.
